# A Bridge Splint for Desperate Cases of Pyorrhea

**Published:** 1901-11-15

**Authors:** W. B. Ames

**Affiliations:** Chicago, Ills.


### A BRIDGE SPLINT FOR DESPERATE CASES OF PYORRHEA.

BY DR. W. B. AMES, CHICAGO, ILLS.

Synopsis of a paper read before the National Dental Association, August 8, 1901, at Milwaukee, Wis.

By desperate cases the author refers to those cases where the teeth have loosened because the periosteum and alveolar bone has been so extensively absorbed that there is no prospect of ever restoring them to a stable condition, except by some means of tying several of the teeth together, by ligatures, bands or splints. The ligatures even when confined to position by means of the various cement propositions are entirely useless, as they are unreliable and short-lived at best, and difficult to keep in a sanitary condition.

The essayist illustrated his idea by treating an ideal case. It was a case where the lower incisors had been badly affected by pyorrhea and the cuspids not so seriously, but still there had been considerable loss of peridental support. The crowns of these six teeth were sawed off at the cervical portion, or at the point of the gingival attachment in health. The pulps all removed and their roots filled, and the soft and adjacent bone tissues brought to as good a condition of health as possible. Then gold bands a trifle more than an eighth of an inch wide were made and adapted to the stump of each tooth, allowing the band to embrace the root for one-sixteenth of an inch and to project above the end of the stump for a sixteenth of an inch; a plate of gold was then fitted inside of the band and to the end of the root: a post was then set into the root canal through a hole punched in the cap and the whole soldered together, making a cap and post like the one used in making a Richmond porcelain crown, except for the band projecting above the cap or end of the root. The post is also permitted to project above the root end an eighth of an inch or more if practicable. The natural teeth crowns which have been sawed off then have their pulp chambers drilled out and they are fitted into the band socket and to the end of the post. The gold bands and posts are then fitted to place on the stumps of the roots and an impression removes them so that they may be united by soldering. When soldered together the bridge is finished and cemented to the stumps. The crowns are then adjusted each to its proper place and cemented to the bridge. The result is artistic and if well done a substantial-appearing piece of work. The gold bands showing at the cervical borders and having the appearance of well-fitting single bands. The porcelain crowns can be used when desired by using tube or countersunk teeth.

The claim for this method is that the roots of the teeth are held more rigidly together and treatment can be applied to restore lost structure and a normal condition of the gum and process. And the pulps having been removed from the teeth there is none of the usual hypersensitiveness of the teeth or uncontrollable neuralgias.

### DISCUSSION.

Dr. N. S. Hoff, of Ann Arbor, Mich.: The operation is admirably done and I have no criticisms to make on the mechanical construction of the bridge. The method is so radical that it seems destructive rather than conservative. The idea of destroying the pulps in so many teeth and the removal of their crowns seems so contrary to good practice that I am not sure but that it is a mistake. This method if adopted permanently mutilates the natural teeth and compels the wearing of the bridge permanently even should the roots and their sockets be brought to a condition of perfect health and function. A splint made in this manner, because it secures rigidity of the roots would unquestionably prove beneficial, while the present destructive condition existed, but would be a great hindrance to the natural function of the teeth after a cure had been effected. By banding the six teeth together an artificial condition is permanently produced. The teeth can not occlude properly, and the normal function of individual teeth is under continued artificial restraint. The tactil sense of the individual tooth is lost. The unrestrained motion of the individual teeth in their sockets is lost and with this goes the nutritive function of the peridental structures. So that I am not sure that the idea of permanently disabling six important teeth, for instance, is a justifiable proceedure. There are other methods of securely fixing the teeth, which do not involve their mutilation, and should the teeth and surrounding tissues be restored to a normal condition the fixture may be removed and no unpleasant results are left to jeopardize the treatment. As to the matter of destroying the pulps to prevent excessive sensitiveness, I can't think this is often made necessary.

Dr. W. F. Litch, of Philadelphia: For the case as presented I would consider such a proceedure excessively radical. There are simpler appliances which are surely as effective as this can be, for holding the teeth rigidly in their sockets. I have had good results from adapting a metal splint to the lingual surfaces with gutta-percha cement and then ligaturing the teeth firmly and permanently to it with wire ligatures. The metalic bands properly fitted to the teeth covering the exposed parts of the root may have a therapeutic value. They surely would in overcoming excessive sensitiveness. I can't commend the principle of uniting so many teeth together in a bridge. The life of the teeth will unquestionably be shortened.

Dr. S. H. Guilford, of Philadelphia: As an expedient in cases of extreme desperation this method would doubtless have a valid claim. I don't like the idea of destroying so many pulps if it is at all practicable to save the teeth by any other proceedure, not even if it should make it possible to save a few loose teeth thereby. I should prefer a temporary or removable splint and I have no doubt patient would also. My method usually is to band the teeth only upon the enamel in such a way as to show the metal as little as possible. It is sometimes necessary to band the teeth not only during treatment, but permanently. When the latter is necessary it should be done judiciously and the bands should be of sufficient size as to give the required strength and endurance.

Dr. J. D. Patterson, of Kansas City, Mo.: In all cases where the teeth are badly affected with pyorrhea and sensitive, it is a proper practice to destroy their pulps. I always do so. The use of silk or other fibrous ligature is exceedingly unsatisfactory and are seldom justifiably used. Where there is any occasion to employ a splint at all, it should be made and adapted as a permanent appliance, as I do not believe such conditions ever can be restored to such a condition of health as to permit of its removal without injury. A method I have used frequently with good results is to swedge a gold plate to fit the lingual surfaces, of the incissor teeth for instance, upon a metal die. Then fit it to the teeth in the mouth and fasten by cement and pins soldered to the splint, which pass through holes drilled through the teeth. from the labial to the lingual surfaces. These pins are then cut off on the labial surfaces of the incisor and riveted. The pins used are made of No. 16 soft pure gold wire.

DR. J. A. STUBBLEFIELD, of Nashville, Tenn.: As I understand it the essayist only advocates this method for teeth that are practically hopeless, so far as the usual method of treatment is concerned. For such cases I think we might be justified in going to the radical extreme which he advocates. I do not, however, like the idea of cutting off the natural crowns when they are not carious, to be substituted by artificial ones.

DR. G. NEWKIRK, of Los Angeles, Cal.: The method advocated by the essayist would be all right for him to use because he has not only skill but judgment; but when advocated for general use, I fear it would be likely to be misused oftener than it would be applied to advantage. I can, however concur in the idea of destroying the pulps, as I believe the irritable pulps oftentimes have much to do with prolonging the disease.

The use of fibrous ligatures should be condemned for the reason that they lack permanence.

Dr. Bogue, of New York, thanked the essayist for his unique and valuable contribution. It has much to commend. Can not, however, understand why Dr. Ames should care to use the natural crowns rather than the porcelain ones now obtainable in all shades and sizes.

Dr. V. H. Jackson: The essayist advocated the replantation of an ex-foliated tooth in this method. I would not do that, but by using some other tooth an attachment would result which would be satisfactory.

Dr. Ames, in closing, objected to the suggestion that the operation could not be called conservative, for it was essentially so, since it was only advocated for such conditions as were hopeless by other proceedures. I regard the use of ligatures and bands, which can not be accurately fitted, as useless, and an imposition on the patient, as they will lead to failure surely. In replanting ex-foliated roots I do not expect peridental attachment. I expect the root to be held firmly in place and to serve its purpose of completing the arch for esthetic purposes.

